# Classifying Lupus Nephritis: An Ongoing Story

**DOI:** 10.1155/2014/580620

**Published:** 2014-12-08

**Authors:** Saba Kiremitci, Arzu Ensari

**Affiliations:** Pathology Department, Medical School of Ankara University, Sihhiye, 06100 Ankara, Turkey

## Abstract

The role of the renal biopsy in lupus nephritis is to provide the diagnosis and to define the parameters of prognostic and therapeutic significance for an effective clinicopathological correlation. Various classification schemas initiated by World Health Organization in 1974 have been proposed until the most recent update by International Society of Nephrology/Renal Pathology Society in 2004. In this paper, we reviewed the new classification system with the associated literature to highlight the benefits and the weak points that emerged so far. The great advantage of the classification emerged to provide a uniform reporting for lupus nephritis all over the world. It has provided more reproducible results from different centers. However, the studies indicated that the presence of glomerular necrotizing lesion was no longer significant to determine the classes of lupus nephritis leading to loss of pathogenetic diversity of the classes. Another weakness of the classification that also emerged in time was the lack of discussions related to the prognostic significance of tubulointerstitial involvement which was not included in the classification. Therefore, the pathogenetic diversity of the classification still needs to be clarified by additional studies, and it needs to be improved by the inclusion of the tubulointerstitial lesions related to prognosis.

## 1. Introduction

Systemic lupus erythematosus (SLE) is a systemic autoimmune disease with a common finding of renal involvement which is related to high incidence of mortality and morbidity [1]. The role of renal biopsy is critical in the management of patients with lupus nephritis (LN). All renal compartments including glomerular, tubulointerstitial, and vascular components may be injured by the disease; however, the term “lupus nephritis” is mainly used to define the immune complex-mediated glomerulonephritis. Lupus nephritis may present in diverse clinical manifestations as well as different histological patterns of renal injury. Thereby, various classification schemas for LN initiated by World Health Organization (WHO) in 1974 [2] have been proposed until the most recent update by International Society of Nephrology/Renal Pathology Society (ISN/RPS) in 2004 [3–5]. The classes included in the classification schemas were designed according to the different morphologic patterns of glomerular injury and their prognostic relevance.

In this review, we shall focus on the modifications in LN classification and summarize the most recent 2004 ISN/RPS classification related to its strengths and weaknesses.

## 2. Evolving Process of LN Classification Schemas

Inception of routine performance of renal biopsy in the 1950s, advances in immunofluorescence microscopy (IFM), and application of electron microscopy (EM) in the 1960s together with the recognition of immune-mediated mechanisms of glomerular injury revealed diverse patterns of renal damage during the course of SLE. Initial approach in 1964 categorized renal biopsy findings into three groups: focal segmental glomerulitis, diffuse proliferative glomerulonephritis, and membranous glomerulopathy [6]. Following identification of mesangial lesions in the 1970s, the first published classification of LN based on data from clinicopathologic characterization was formulated by Pirani and Pollak in New York under the auspices of WHO [2]. This basic classification schema included the classes of normal glomeruli (class I), pure mesangial disease (class II), focal proliferative glomerulonephritis (class III), diffuse proliferative glomerulonephritis (class IV), and membranous glomerulonephritis (class V). Because the distinction between the proliferative classes was not detailed by quantitative guidelines, there appeared inconsistency in referring the classes. The classification was modified by the International Study of Kidney Disease in Children and WHO in 1982 [7]. Main modification was related to the class III category that “focal proliferative glomerulonephritis” was replaced by “focal segmental glomerulonephritis” which indicated particularly focal necrotizing glomerular lesion. Class IV remained the same without indicating the differentiating quantitative criteria (proportion of injured glomeruli) from class III LN. Subdivisions were introduced according to the existence of active necrotizing and/or sclerosing lesions for proliferative classes; moreover, class V “membranous glomerulonephritis” was subdivided according to the concomitance of the other classes, and a new category “advanced sclerosing glomerulonephritis” was also added to the classification schema as class VI. However, neither a precise criterion for overlapping cases nor the required percentage of sclerotic glomeruli for class VI was mentioned. The diversity of subdivisions, the handling of overlap cases, and the inadequacy of the strict criteria differentiating the classes made the classification impracticable for both the pathologists and the clinicians, so that most of the pathologists continued to use the previous classification. Over time, the debate focused on the segmental glomerular fibrinoid necrosis, which is also the characteristic glomerular lesion of systemic vasculitis. Studies revealing worse prognosis for diffuse segmental necrotizing glomerulonephritis led another revision in 1995 where a subcategory “subclass III ≥50%; severe segmental glomerulonephritis” was suggested [8]. While some authors suggested categorizing segmental necrotizing lesion in subclass III or IV according to the proportion of the involved glomeruli, others insisted on emphasizing segmental necrotizing lesion as the defining feature of subclass III independent of the proportion of affected glomeruli and stressed the presence of a different pathogenetic mechanism similar to nonimmune pathogenesis in systemic vasculitis [9]. Subdivisions of membranous glomerulonephritis (class V) were also controversial that some authors suggested classifying the overlap cases (membranous lesion + proliferative lesion) in subclass IV instead of subclass V, due to the significant role of proliferative glomerulonephritis in renal outcome. However, this approach was incompatible with the clinical aspects as persistent proteinuria following the treatment is a manifestation of membranous glomerulopathy.

## 3. ISN/RPS Lupus Nephritis Classification System

Ongoing inconsistencies and ambiguities of the classification schemas made life widely difficult for both pathologists and clinicians. The goal of the renal biopsy protocol was not only to provide the diagnosis, but also to define the parameters of prognostic and therapeutic significance for an effective clinicopathologic correlation. In 2002, under the auspices of ISN/RPS, a new classification system was proposed by a consensus conference held at Columbia University, New York, with the participants: renal pathologists, nephrologists, and rheumatologists. It was aimed at providing a common reporting system for LN which would lead to effective communication of pathologic findings in every sense. Standardization of the biopsy reports from different centers was of first priority; thus, all the definitions, differentiating criteria, and details remaining in suspense were reviewed, and clear and strict criteria were determined to reduce the interobserver variation and to form a uniform reporting system. The clinicopathologic experience of previous WHO classification schemas and the emerging nonimmune mediated pathogenetic mechanisms were considered to achieve a more reproducible classification. Like the previous classification schemas, ISN/RPS classification system was also based on glomerular pathology. It was published by multiple journals in 2004 to promote the widespread use of this classification all over the world [3–5].

The classification pointed the basic approach to the renal biopsy in the following items.

- The renal biopsy should include minimum 10 glomeruli to exclude the existence of focal lesions [10].
- The renal biopsy should be studied by IFM including IgG, IgA, IgM isotypes, Kappa and Lambda light chains, and C3 and C1q complement components. For the diagnosis of LN dominant IgG, C3 and in most instances C1q deposits are absolutely required and variable codeposits of IgA and IgM are also compatible with the diagnosis.
- The lack of EM should not prevent the pathologist from making LN diagnosis; however, for some essential cases, storage of a sample of renal cortical tissue is proposed [11, 12].

In fact, the 2004 ISN/RPS classification was formulated in parallel to the original WHO classification in basic approach; however, it comprised distinct qualitative and quantitative modifications in individual classes.

## 4. What is New in 2004 ISN/RPS Lupus Nephritis Classification System?

**Figure 1:**
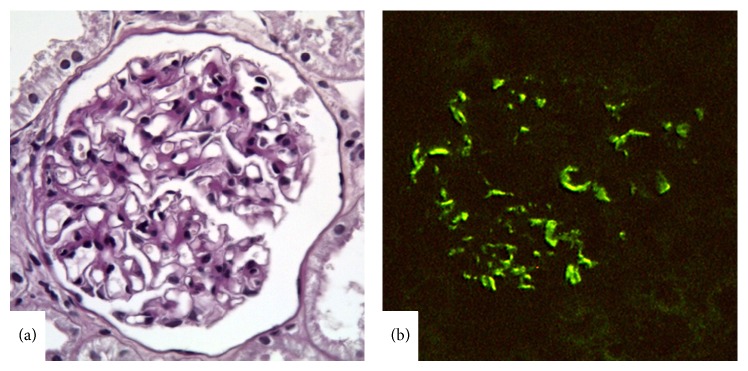
Lupus nephritis class I. (a) A normal appearing glomerulus with no mesangial alteration (periodic acid-Schiff stain, ×400). (b) Mesangial IgG deposition by IFM (×400).

**Figure 2:**
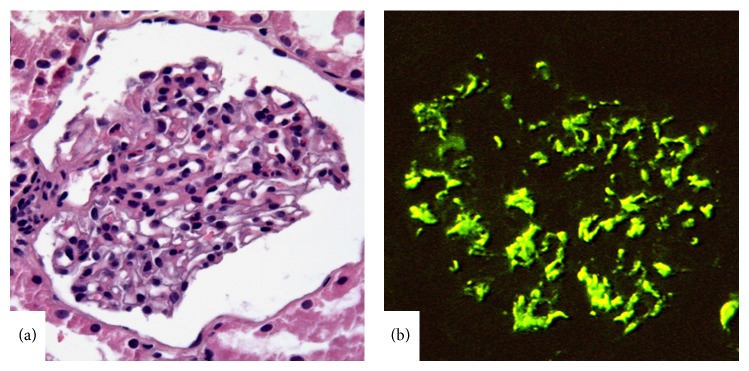
Lupus nephritis class II. (a) Moderate mesangial hypercellularity in the glomerulus (hematoxylin-eosin, ×400). (b) Diffuse and strong mesangial IgG deposition by IFM (×400).

(i) Initially, because EM is not available for all centers practicing renal biopsy routine, the definitions for diagnostic terms were adapted to LM and IFM findings to provide the widespread uniformity of renal biopsy reports around the world (Table 1).

(ii) The category of WHO classification “normal glomeruli (by LM, IFM, and EM)” was excluded from the LN classification. These patients are not included in the daily practice of nephrologists and calling a fully normal renal biopsy as nephritis caused debate; and the category was eliminated with consensus. Class I was replaced by the “minimal mesangial LN” which defines normal glomeruli by LM and mesangial immune deposits by IFM or EM (Figure 1).

(iii) The division of class II LN according to the degree of mesangial hypercellularity in WHO classification had no clinical relevance, so that Class II LN was defined to comprise mesangial hypercellularity and/or matrix expansion of any degree in addition to mesangial immune deposits and termed “mesangial proliferative LN” (Figure 2). No glomerular lesion other than mesangial alterations is compatible with class II, and the immune deposits are restricted to the mesangium by IFM or EM and are inconspicuous by LM. Exceptional cases comprising rare isolated small immune deposits in the peripheral capillary walls by IFM or EM were also included in class II in ISN/RPS classification. However, any subendothelial or subepithelial deposits detectable by LM are not compatible with class II whether or not there is endocapillary proliferation.

(iv) Modifications related to proliferative classes of WHO classification: potential glomerular lesions except for mesangial alterations seen in LN were listed and were individually specified as active or chronic lesions to provide the optimal correlation with the clinical findings (Table 2). These lesions include various endocapillary and extracapillary changes and the existence of any of these lesions alone or together was attributed to the higher classes of LN.

The first common modification in class III and class IV LN was to remove the term “proliferative” from the nomenclature of these classes. Thus, the new nomenclature “Focal LN” for class III and “Diffuse LN” for class IV could cover the heterogeneous phenotype including isolated extracapillary proliferation (i.e., crescents), membranoproliferative features, and subendothelial wire loop deposits without proliferation other than classical endocapillary proliferation [13].

Class III and class IV were designed as classes sharing the same diversity of glomerular lesions but differing mainly in the severity of the lesions. If the percentage of glomeruli exhibiting any of the glomerular lesions was <50% or ≥50%, the case was classified as class III or class IV, respectively. While determining the class of LN, making the right decision of the percentage of involved glomeruli comes into prominence. Because this issue was not handled in detail in prior classifications, interobserver variability was significant in rating the sclerotic glomeruli (focal or global glomerulosclerosis) that while some of the pathologists considered the sclerotic glomeruli in assessing the extent of the lesions, others ignored them. However, the ISN/RPS classification referred to the sclerotic glomerular lesions as chronic sequel of previous endocapillary proliferation, necrosis, or crescents and guided to include the sclerotic glomerular lesions in the assessment of total affected glomeruli. Besides, attention must be paid in order to not misinterpret ischemic obsolescence of the glomeruli as a scar of previous proliferative lesion.

The last common modification of classes III and IV was to use simple letters for active and chronic lesion subcategories, proposed as (A) and (C), respectively, instead of numbered or lettered subcategories hard to keep in mind.

**Figure 3:**
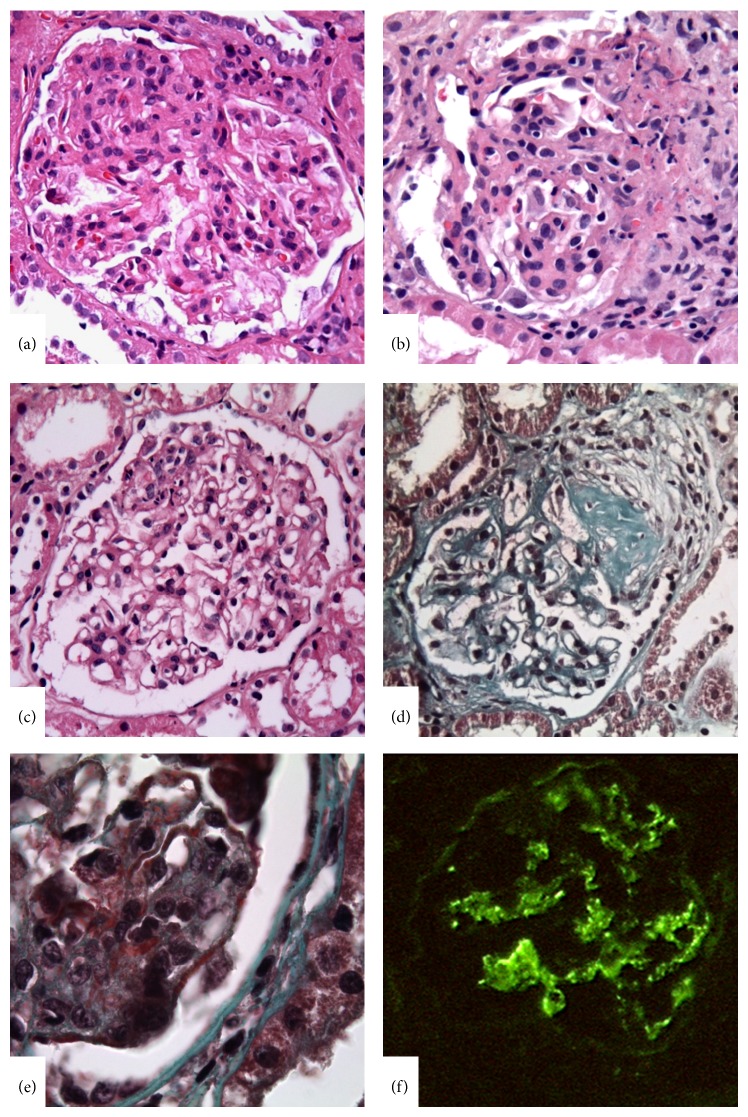
Lupus nephritis class III. (a) Segmental endocapillary hypercellularity with substantial luminal reduction. (b) Endocapillary hypercellularity with fibrinoid necrosis and cellular crescent formation. (c) Karyorrhexis in a segment of a glomerulus. (d) Segmental sclerosis of a glomerulus. (e) Segmental subendothelial deposits by light microscopy as fuchsinophilic deposits in Masson Trichrome stain. (f) IgG immune deposits in glomerular capillary wall in segmental distribution and also accompanying mesangial deposits (IFM, ×400).

**Figure 4:**
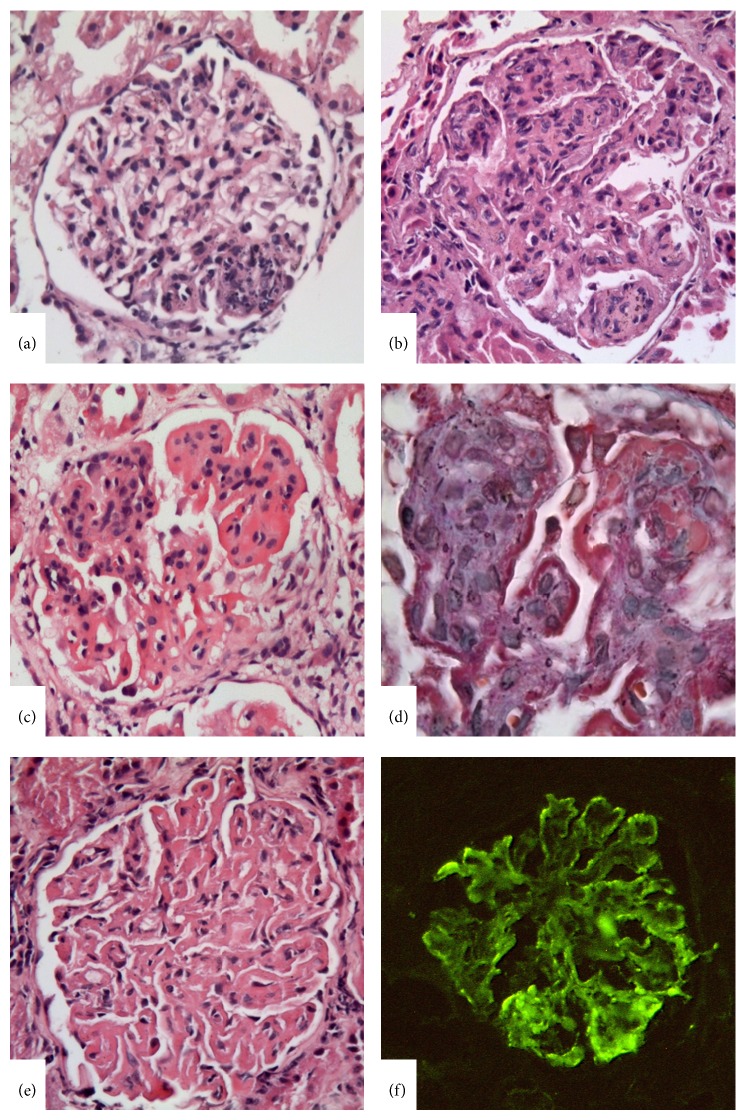
Lupus nephritis class IV. (a) Class IV-S case with segmental endocapillary proliferation. The lesion was diffuse in the biopsy (not shown in the picture). (b) Global endocapillary proliferation with leukocyte infiltration. (c) Endocapillary proliferation with widespread wire loop appearance in the glomerular capillary wall indicator of subendothelial immune deposits by light microscopy. (d) Reflection of the subendothelial deposits as fuchsinophilic deposits in Masson Trichrome stain. (e) Global wire loop appearance without mesangial or endocapillary cellular proliferation. This finding was diffuse in the biopsy (not shown in the picture). (f) Significant immune deposit overload in glomerular capillary wall by IFM (×400).

According to the ISN/RPS classification, the glomerular lesions could be either segmental or global in both class III and class IV; however, it is also stressed that the focal lesions of class III tend to be segmental, and diffuse lesions of class IV tend to be global. The diversity of the glomerular lesions was demonstrated in Figure 3 and Figure 4. The classification defined subendothelial immune deposits usually in a segmental distribution as well as diffuse mesangial deposits for class III LN but also pointed to the rare vasculitis-like lesions characterized by segmental capillary necrosis without any endocapillary proliferation and immune deposits [14, 15]. For class IV LN, diffuse subendothelial immune deposits with or without mesangial deposits were defined characteristic, although extensive subendothelial deposits with little or no proliferation (widespread wire loops), and the common finding of scattered subepithelial deposits may also be compatible with class IV.

One of the major modifications of the recent classification was subdivision of class IV LN as class IV-S (diffuse segmental LN) and class IV-G (diffuse global LN), according to the majority of segmental and global glomerular lesions, respectively. It was aimed at exhibiting both the potential pathogenetic diversity regarding the studies suggesting different pathogenetic mechanisms for distinct cases with segmental fibrinoid necrosis without endocapillary proliferation and subendothelial deposits [14, 15] and the different outcomes for diffuse segmental glomerulonephritis and diffuse global glomerulonephritis which were stressed in some studies [9, 16].

**Figure 5:**
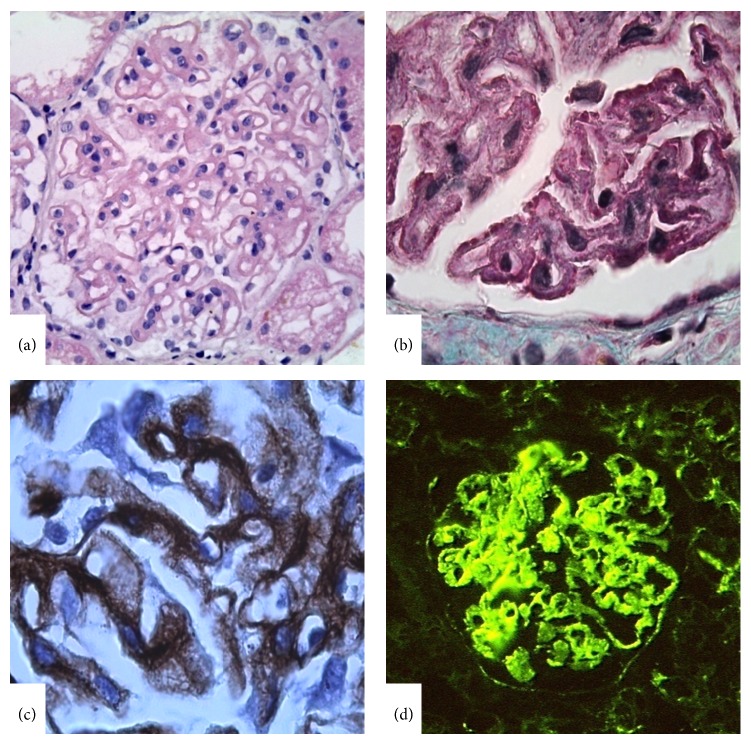
Lupus nephritis class V. (a) Significant glomerular capillary wall thickening in global distribution. (b) Subepithelial fuchsinophilic deposits revealed in Masson Trichrome stain. (c) “Spikes” in glomerular capillary wall indicator of membranous nephropathy by Periodic Schiff-Methenamine Silver Stain. (d) Continuous subepithelial IgG deposits in the glomerular capillary wall (IFM, ×400).

(v) One of the major modifications of the ISN/RPS classification was related to class V membranous LN. Definition of the class and elimination of the confusing subgroups were achieved by composing clear distinct criteria. Class V LN was defined as membranous LN with global or segmental continuous granular subepithelial immune deposits. It was suggested that the existence of subendothelial deposits by LM and/or active or chronic glomerular lesions requires using combined diagnosis of LN as “classes III and V” or “classes IV and V” according to the extent of the glomerular lesions. The strict criteria for the diagnosis of “combined case” were defined as subepithelial deposits involving >50% of the tuft of >50% of the glomeruli (Figure 5). Each class was to be reported in the diagnostic line to avoid the miscommunication and to highlight the importance of proliferative lesion which will determine the clinical outcome.

(vi) The last modification was the definition of class VI (advanced-stage LN) which was not mentioned in the previous classifications at all. If global glomerulosclerosis concerning ≥90% of the total glomeruli occurs and clinical or pathologic evidence supports the LN etiology and also there is no active lesion, it is attributable to class VI LN.

*Recommendation for Reporting*. The ISN/RPS classification stated a number of recommendations for reporting renal biopsy in a patient with LN including a detailed description of all of the findings by LM, IFM, and EM in an organized manner, followed by a diagnostic line summarising the class of LN, percentage of glomeruli with active and/or sclerotic lesions, grade of tubular atrophy, interstitial fibrosis, and interstitial inflammation. The use of activity and chronicity index [17] was also encouraged, but the superiority of the detailed description of the activity and chronicity parameters were stressed. The classification also specified the importance of vascular damage and indicated that this should be included in the diagnostic line.

*What Happened after the ISN/RPS Classification*? In the advancing process, the mission of this recent classification was highly achieved that elimination of the normal category (WHO class I) and removal of subcategories of class V LN provided a better clinical correlation; further the use of simple designations (A) and (C) for active and chronic lesions and frequently repeated 50% cut-off in the classification simplified the reporting and an effective communication of the pathologist and the clinicians was established. In a number of studies, interobserver and intraobserver reproducibility of the specification of the classes were studied by comparing WHO 1995 and ISN/RPS 2004 classification systems for LN [18–20]. The studies revealed much higher consensus in the judgment of classes in ISN/RPS classification system compared to WHO classification. However, a new report studying the interobserver agreement of classes indicated a poor agreement in terms of recognizing class III/IV lesions [21]. We summarized the findings of interobserver validation studies in the literature in Table 3. Standardization of the definitions for each class with clear discriminating criteria and a well-defined approach in the handling of sclerotic glomeruli improved both intraobserver and interobserver reproducibility and enabled reliable comparison of different cases from various centers [13, 22–26]. Moreover, the morphologic parameters indicative of prognostic value and outcome contributed to the medical literature. Thus, the great advantage of this classification appeared to provide a uniform reporting for LN constituting the backbone of prospective studies related to therapy and prognosis [25].

The benefits and the concerns of ISN/RPS classification were summarized in Table 4.

## 5. Weak Points of the Classification

However, with the adaptation of the classification in daily routine, weaknesses have also emerged.

- While determining the activity (A) and chronicity (C) designations for glomerular lesions, it was not clear how many active and chronic lesions were needed. It was suggested that a single glomerular lesion with any feature of activity and a single glomerular sclerotic lesion as a scar of glomerulonephritis were enough to designate “A” and “C,” respectively [23, 25]. However, the degree of activity is important to decide on a more aggressive therapy. By the use of ISN/RPS classification, interobserver reproducibility in the assessment of disease activity improved significantly, while the assessment of chronicity remained suboptimal [19, 20]. Recent studies have also indicated interstitial inflammation as one of the significant independent risk factors for renal outcome [27–29] in lupus nephritis. In addition to chronic glomerular lesions, the chronicity index in the judgment of a renal biopsy is fundamentally based on the chronic tubulointerstitial lesions [17, 30]. Tubular atrophy and interstitial fibrosis are the main parameters related to renal function and responsiveness to therapy [31]. Not only proliferative classes of LN but also nonproliferative classes may comprise remarkable interstitial inflammation, and it is stressed that the persistence of interstitial inflammation after therapy may predict the renal failure [27, 28]. However, the ISN/RPS classification system is based solely on the glomerular involvement with no reference to tubulointerstitial lesions. As tubular atrophy, interstitial fibrosis, and inflammation are not sufficiently quantified together with active and sclerotic lesions in the diagnostic line, the use of activity and chronicity index is still needed in the reporting.
- The significance of the subendothelial deposits in determining the class of LN was not sufficiently stressed in the classification and there emerged cases with no subendothelial deposits or endocapillary lesions visible by LM, but substantial subendothelial deposits by IFM or EM. Markowitz and D'Agati [23] suggested handling such a case according to the distribution of the subendothelial deposits; they offered to classify them as class III if the subendothelial deposits involved <50% of the glomeruli or class IV if they involved ≥50% of the glomeruli. Another controversial point with the subendothelial deposits was the existence of scattered small subendothelial deposits by IFM or EM in class II LN. The debate focused on the potential of these exceptional cases to progress to a more severe or higher class of LN, so that including these cases in class II and adding a comment in the report indicating the potential for progression to a higher class and close follow-up were recommended [13, 14, 32].
- The most significant controversy focused on the potential outcomes of class IV-S and class IV-G subdivision. Multiple centers could examine the distinction of these two subclasses in terms of pathologic findings, clinical, serologic, and prognostic parameters, and treatment modalities [9, 16, 18, 33]. The clinical and serologic data and also clinical outcomes did not significantly differ between class IV-S and class IV-G LN in most of the studies; however, pathologic differences were commonly reported such as extensive fibrinoid necrosis and less prominent immune deposits more likely for class IV-S and significant subendothelial immune deposits more likely for class IV-G [9, 18]. Thus, the authors suggested a different mechanism for class IV-S that is similar to “pauci-immune” vasculitic glomerulonephritis due to the minority of immune deposit load and intensity of fibrinoid necrosis. However, there were also studies showing potential interconversion between these classes in repeat biopsies and refuting the different pathogenesis hypothesis [16, 33]. Most recently, the significance of the segmental glomerular lesion came into prominence particularly related to the severe segmental glomerulonephritis which was previously referred to in WHO classification as “class III ≥50% LN.” In WHO classification, severe segmental glomerulonephritis (class III ≥50%) had different morphologic characteristics and serologic findings compared to class IV of WHO classification. However, in ISN/RPS classification, this distinct group is handled partly under class IV, some in class IV-S, and some in class IV-G. The authors suggested that the pathogenetic and prognostic implications of the segmental lesion of LN were lost by the ISN/RPS classification [34, 35].

## 6. Conclusion

In conclusion, the ISN/RPS classification provided significant advances in the handling of renal biopsies of SLE patients. The most striking advantage of the classification is the high interobserver and intraobserver reproducibility resulting from a uniform reporting system used around the world. Thus, different centers could reliably compare the results of their studies and more reproducible results could be achieved in terms of both pathogenetic relevance and renal outcome. The weak points of the classification, on the other hand, included the loss of pathogenetic diversity of the classes due to the fact that the presence of glomerular necrotizing lesion was no longer significant to determine the classes of lupus nephritis. Secondly, the classification does not sufficiently stress the involvement of nonglomerular compartments such as the tubulointerstitium which may also be related to the prognosis. Hereby, the classification needs to be improved by the inclusion of tubulointerstitial lesions and the pathogenetic diversity of the classes still needs to be clarified with additional studies in order to interpret the potential pathogenetic relevance of the classes.

## Figures and Tables

**Table 1:** Definitions for diagnostic terms according to the 2004 ISN/RPS lupus nephritis classification.

Diffuse	A lesion involving most (≥50%) glomeruli

Focal	A lesion involving <50% of glomeruli

Global	A lesion involving more than half of the glomerular tuft

Segmental	A lesion involving less than half of the glomerular tuft (i.e., at least half of the glomerular tuft is spared)

Mesangial hypercellularity	At least three mesangial cells per mesangial region in a 3-micron thick section

Endocapillary proliferation	Endocapillary hypercellularity due to increased number of mesangial cells, endothelial cells, and infiltrating monocytes, causing narrowing of the glomerular capillary lumina

Extracapillary proliferation or cellular crescent	Extracapillary cell proliferation of more than two cell layers occupying one-fourth or more of the glomerular capsular circumference

Karyorrhexis	Presence of apoptotic, pyknotic, and fragmented nuclei

Necrosis	A lesion characterized by fragmentation of nuclei or disruption of the glomerular basement membrane, often associated with the presence of fibrin-rich material

Hyaline thrombi	Intracapillary eosinophilic material of a homogeneous consistency which by immunofluorescence has been shown to consist of immune deposits

Proportion of involved glomeruli	Intended to indicate the percentage of total glomeruli affected by lupus nephritis, including the glomeruli that are sclerosed due to lupus nephritis but excluding ischemic glomeruli with inadequate perfusion due to vascular pathology separate from lupus nephritis

**Table 2:** Active and chronic glomerular lesions specified by the 2004 ISN/RPS lupus nephritis classification.

Active lesions:	
Endocapillary hypercellularity with or without	
leukocyte	
infiltration and with substantial luminal reduction	
Karyorrhexis	
Fibrinoid necrosis	
Rupture of glomerular basement membrane	
Crescents, cellular, or fibrocellular	
Subendothelial deposits identified by light microscopy	
(wire loops)	
Intraluminal immune aggregates (hyaline thrombi)	
Chronic lesions:	
Glomerular sclerosis (segmental/global)	
Fibrous adhesions	
Fibrous crescents	

**Table 3:** Interobserver validation studies in lupus nephritis.

	Number of cases	Methodology and results
		Comparison of pathologic diagnoses between two pathologists
Yokoyama et al., 2004 [18]	60	Fisher exact test:
		WHO 1995 versus ISN/RPS: 83% versus 98%, *P*: 0.084

		*κ* values^*^:
Furness and Taub, 2006 [19]	20	WHO 1995 versus ISN/RPS: 0.44 versus 0.53, *P*: 0.002
		Acute changes: 0.39
		Chronic changes: 0.35

		ICC^**^:
		WHO 1995 versus ISN/RPS: 0.182 versus 0.414
Grootscholten et al., 2008 [20]	126	Glomerular lesions: 0.439–0.950
		Tubulointerstitial lesions: 0.418–0.514
		Activity index: 0.716
		Chronicity index: 0.494

Wilhelmus et al., 2014 [21]	30	*κ* values/ICC^***^:
	(microphotographs)	presence of class III/IV lesion: 0.39

^*^ *κ* values with 95% confidence interval were calculated to represent the level of interobserver agreement (0 = no agreement and 1 = perfect agreement).

^**^ICC (intraclass correlation coefficient) is an index of concordance that indicates the degree of agreement: >0.8: excellent; 0.6–0.8: good; 0.4–0.6: moderate; <0.4: poor concordance.

^***^Interobserver agreement among nephropathologists was studied. Glomeruli pictures were shared with 360 members of Renal Pathology Society and they were asked whether glomerular lesions were present and compatible with class III or IV.

**Table 4:** Benefits and concerns of the ISN/RPS classification of LN.

Benefits:	

(1) better clinical correlation	
(2) effective communication between pathologists and clinicians	
(3) improvement in reproducibility	
(4) uniformity in reporting	
(5) reliable outline for prospective studies	

Concerns:	

(1) loss of pathogenetic diversity of the classes	
(2) failure to emphasize prognostic significance of	
tubulointerstitial lesions	
(3) unclarified role for subendothelial deposits in determining	
the classes	

## References

[1] Duarte C., Couto M., Ines L., Liang M. H., Lahita R. G., Tsokos G., Buyon J., Koike T. (2011). Epidemiology of systemic lupus erythematosus. *Systemic Lupus Erythematosus*.

[2] McCluskey R. T., Sommers S. C., East Norwalk C. T. (1975). Lupus nephritis. *Kidney Pathology Decennial 1966–1975*.

[3] Weening J. J., D'Agati V. D., Schwartz M. M., Seshan S. V., Alpers C. E., Appel G. B., Balow J. E., Bruijn J. A., Cook T., Ferrario F., Fogo A. B., Ginzler E. M., Hebert L., Hill G., Hill P., Jennette J. C., Kong N. C., Lesavre P., Lockshin M., Looi L. M., Makino H., Moura L. A., Nagata M. (2004). International Society of Nephrology Working Group on the Classification of Lupus Nephritis; Renal Pathology Society Working Group on the Classification of Lupus Nephritis. The classification of glomerulonephritis in systemic lupus erythematosus revisited. *Kidney International*.

[4] Weening J. J., D’Agati V. D., Schwartz M. M. (2004). The classification of glomerulonephritis in systemic lupus erythematosus revisited. *Journal of the American Society of Nephrology*.

[5] Weening J. J., D'Agati V. D., Schwartz M. M. (2004). Erratum in “The classification of glomerulonephritis in systemic lupus erythematosus revisited”. *Kidney International*.

[6] Pollak V. E., Pirani C. L., Schwartz F. D. (1964). The natural history of the renal manifestations of systemic lupus erythematosus. *The Journal of Laboratory and Clinical Medicine*.

[7] Churg J., Sobin L. H. (1982). *Renal Disease: Classification and Atlas of Glomerular Disease*.

[8] Churg J., Bernstein J., Glassock R. J. (1995). *Renal Disease: Classification and Atlas of Glomerular Diseases*.

[9] Najafi C. C., Korbet S. M., Lewis E. J., Schwartz M. M., Reichlin M., Evans J. (2001). Significance of histologic patterns of glomerular injury upon long-term prognosis in severe lupus glomerulonephritis. *Kidney International*.

[10] Corwin H. L., Schwartz M. M., Lewis E. J. (1988). The importance of sample size in the interpretation of the renal biopsy. *American Journal of Nephrology*.

[11] Pirani C. L., Olesnicky L., Hayslett J. P., Hardin G. A. (1983). Role of electron microscopy in the classification of lupus nephritis. *Advances in Systemic Lupus Erythematosus*.

[12] Herrera G. A. (1999). The value of electron microscopy in the diagnosis and clinical management of lupus nephritis. *Ultrastructural Pathology*.

[13] Seshan S. V., Jennette J. C. (2009). Renal disease in systemic lupus erythematosus with emphasis on classification of lupus glomerulonephritis advances and implications. *Archives of Pathology and Laboratory Medicine*.

[14] D’Agati V., Jennette J. C., Olson J. L., Schwartz M. M., Silva F. G. (1998). Renal disease in systemic lupus erythematosus, mixed connective tissue disease, Sjogren’s syndrome, and rheumatoid arthritis. *Heptinstall's Pathology of the Kidney*.

[15] Ferrario F., Napodano P., Giordano A., Gandini E., Boeri R., D'Amico G. (1992). Peculiar type of focal and segmental lupus glomerulitis: glomerulonephritis or vasculitis?. *Contributions to Nephrology*.

[16] Hill G. S., Delahousse M., Nochy D., Bariéty J. (2005). Class IV-S versus class IV-G lupus nephritis: clinical and morphologic differences suggesting different pathogenesis. *Kidney International*.

[17] Austin H. A., Muenz L. R., Joyce K. M. (1984). Diffuse proliferative lupus nephritis: identification of specific pathologic features affecting renal outcome. *Kidney International*.

[18] Yokoyama H., Wada T., Hara A., Yamahana J., Nakaya I., Kobayashi M., Kitagawa K., Kokubo S., Iwata Y., Yoshimoto K., Shimizu K., Sakai N., Furuichi K. (2004). The outcome and a new ISN/RPS 2003 classification of lupus nephritis in Japanese. *Kidney International*.

[19] Furness P. N., Taub N. (2006). Interobserver reproducibility and application of the ISN/RPS classification of lupus nephritis—a UK-wide study. *American Journal of Surgical Pathology*.

[20] Grootscholten C., Bajema I. M., Florquin S., Steenbergen E. J., Peutz-Kootstra C. J., Goldschmeding R., Bijl M., Hagen E. C., Van Houwelingen H. C., Derksen R. H. W. M., Berden J. H. M. (2008). Interobserver agreement of scoring of histopathological characteristics and classification of lupus nephritis. *Nephrology Dialysis Transplantation*.

[21] Wilhelmus S., Cook H. T., Noel L. H., Ferrario F., Wolterbeek R., Brujin J. A., Bajema I. M. (2014). Interobserver agreement on histopathological lesions in class III or IV lupus nephritis. *Clinical Journal of the American Society of Nephrology*.

[22] Chan T. M. (2005). Histological reclassification of lupus nephritis. *Current Opinion in Nephrology and Hypertension*.

[23] Markowitz G. S., D'Agati V. D. (2007). The ISN/RPS 2003 classification of lupus nephritis: an assessment at 3 years. *Kidney International*.

[24] Markowitz G. S., D'Agati V. D. (2009). Classification of lupus nephritis. *Current Opinion in Nephrology and Hypertension*.

[25] Ortega L. M., Schultz D. R., Lenz O., Pardo V., Contreras G. N. (2010). Lupus nephritis: pathologic features, epidemiology and a guide to therapeutic decisions. *Lupus*.

[26] Hwang J., Kim H. J., Oh J.-M., Ahn J. K., Lee Y. S., Lee J., Kim Y.-G., Huh W.-S., Seo J., Koh E.-M., Cha H.-S. (2012). Outcome of reclassification of world health organization (WHO) class III under international society of nephrology-renal pathology society (ISN-RPS) classification: retrospective observational study. *Rheumatology International*.

[27] Alsuwaida A. O. (2013). Interstitial inflammation and long-term renal outcomes in lupus nephritis. *Lupus*.

[28] Hsieh C., Chang A., Brandt D., Guttikonda R., Utset T. O., Clark M. R. (2011). Predicting outcomes of lupus nephritis with tubulointerstitial inflammation and scarring. *Arthritis Care & Research*.

[29] Yu F., Wu L. H., Tan Y., Li L. H., Wang C. L., Wang W. K., Qu Z., Chen M. H., Gao J. J., Li Z. Y., Zheng X., Ao J., Zhu S. N., Wang S. X., Zhao M. H., Zou W. Z., Liu G. (2010). Tubulointerstitial lesions of patients with lupus nephritis classified by the 2003 International Society of Nephrology and Renal Pathology Society system. *Kidney International*.

[30] Austin H. A., Muenz L. R., Joyce K. M., Antonovych T. A., Kullick M. E., Klippel J. H., Decker J. L., Balow J. E. (1983). Prognostic factors in lupus nephritis. Contribution of renal histologic data. *The American Journal of Medicine*.

[31] Howie A. J., Turhan N., Adu D. (2003). Powerful morphometric indicator of prognosis in lupus nephritis. *QJM*.

[32] Lee S. G., Cho Y. M., So M. W., Kim S. S., Kim Y.-G., Lee C.-K., Yoo B. (2012). ISN/RPS 2003 class II mesangial proliferative lupus nephritis: a comparison between cases that progressed to class III or IV and cases that did not. *Rheumatology International*.

[33] Mittal B., Hurwitz S., Rennke H., Singh A. K. (2004). New subcategories of class IV lupus nephritis: are there clinical, histologic, and outcome differences?. *American Journal of Kidney Diseases*.

[34] Schwartz M. M., Korbet S. M., Lewis E. J. (2008). The prognosis and pathogenesis of severe lupus glomerulonephritis. *Nephrology Dialysis Transplantation*.

[35] Behara V. Y., Whittier W. L., Korbet S. M., Schwartz M. M., Martens M., Lewis E. J. (2010). Pathogenetic features of severe segmental lupus nephritis. *Nephrology Dialysis Transplantation*.

